# Comparative analysis of geotypic variations in the proteome of *Nostoc commune*

**DOI:** 10.1080/15592324.2024.2370719

**Published:** 2024-06-24

**Authors:** Deepti Routray, Arindam Ghatak, Palak Chaturvedi, Linda Petijová, Wolfram Weckwerth, Dajana Ručová, Martin Bačkor, Ingeborg Lang, Michal Goga

**Affiliations:** a Department of Plant Biology, Institute of Biology and Ecology, Faculty of Science, Pavol Jozef Šafárik University in Košice, Košice, Slovakia; b Molecular Systems Biology Lab, Department of Functional and Evolutionary Ecology, University of Vienna, Vienna, Austria; c Vienna Metabolomics Center, University of Vienna, Vienna, Austria; d Department of Genetics, Faculty of Science, Pavol Jozef Šafárik University in Košice, Košice, Slovakia; e Institute of Biotechnology, Faculty of Biotechnology and Food Sciences, Slovak University of Agriculture in Nitra, Nitra, Slovakia

**Keywords:** Cyanobacteria, stress, proteomics, plasticity, LC-MS, AN, Europe

## Abstract

Cyanobacterium *Nostoc commune* is a filamentous terrestrial prokaryotic organism widely distributed, which suggest its high adaptive potential to environmental or abiotic stress. Physiological parameters and proteomic analysis were performed in two accession of *N. commune* with the aim to elucidate the differences of physiological trails between distant geotypes, namely Antarctic (AN) and central European (CE). The result obtained clearly showed that the AN geotype demonstrates elevated levels of total phenols, flavonoids, carotenoids, and phycobiliproteins, indicative of its adaptation to environmental stress as referred by comparison to CE sample. Additionally, we employed LC-MS analysis to investigate the proteomes of *N. commune* from AN and CE geotypes. In total, 1147 proteins were identified, among which 646 proteins expressed significant (up-regulation) changes in both accessions. In the AN geotype, 83 exclusive proteins were identified compared to 25 in the CE geotype. Functional classification of the significant proteins showed a large fraction involved in photosynthesis, amino acid metabolism, carbohydrate metabolism and protein biosynthesis. Further analysis revealed some defense-related proteins such as, superoxide dismutase (SOD) and glutathione reductase, which are rather explicitly expressed in the AN *N. commune*. The last two proteins suggest a more stressful condition in AN *N. commune*. In summary, our findings highlight biochemical processes that safeguard the AN geotype of *N. commune* from extreme environmental challenges, not recorded in CE accession, probably due to less stressful environment in Europe. This study brings the first ever proteomic analysis of *N. commune*, emphasizing the need for additional investigations into the climate adaptation of this species with rather plastic genome.

## 1. Introduction

Cyanobacteria are gram-negative bacteria which are crucial for production of oxygen and maintenance of global carbon cycle through photosynthesis.^1^ Cyanobacteria have promising applications, such as biofuel generation, wastewater treatment, the production of dietary supplements, and the synthesis of bioactive compounds, exhibiting antimicrobial and anti-cancer properties.^2^ Considering their significant contribution to ecology and the environment, they serve as model organism for studying responses to environmental stress with their endosymbiotic association with higher plants, proposing valuable insights into plastid formation in phototrophic organism.^3^

Cyanobacterium *Nostoc commune* forms semi-terrestrial sheet-like colonies, which are widely distributed from tropical to polar environments.^4,5^ They can survive extreme temperatures like frost and drought and can resume metabolic activity when conditions become favorable.^6,7^ Like Antarctica, with extreme environmental conditions, cyanobacteria can endure and proliferate during summer.^8^ In contrast, Europe has a mild and temperate climate suitable for life, plant vegetation and agriculture.^9^

Proteomic analysis using mass spectrometry^10^ is essential for investigating proteins at cellular, tissue or organism level. By scrutinizing protein expressions, responses, and adaptations to environmental stress, proteomic analysis elucidates cellular processes and their regulatory mechanisms. Particularly during prolonged stress, the role of “adaptation proteins” becomes pivotal, revealing cellular mechanisms of stress response.^3^ Proteomic research on cyanobacteria which was started over a decade ago provides insights into protein expression, accumulation, post-translation modifications and interactions surpassing the capabilities of genomics and transcriptomics alone.^11^ Shotgun proteomics, a widely used MS method, involves the characterization of proteins through liquid chromatography – mass spectrometry (LC-MS)^12^ to obtain peptide sequence information. The shotgun LC-MS/MS method identifies proteins in Data-dependent acquisition (DDA) method.^13^ Modern sophisticated LC-MS has considerably improved due to its sensitivity, speed, resolutions and accuracy.^14–16^ Applications of these methods made it possible to investigate proteomes of *Synechocystis* 6803, *Cyanothece, Anabaena*, *Nostoc*.^11^

Investigation on *Nostoc sp*. PCC 7120 under nitrogen starvation highlighted disturbances in major metabolic pathways, confirmed by up-regulation of stress response proteins.^17^ Study of the *N. commune* proteome in response to UV-B stimulation^18^ explained that *N. commune* exhibited different strategies for UV-B shock and acclimation response. However, knowledge about *N. commune* adaptive strategies toward several environmental challenges is very limited.

Hence, the objective of our study was to compare AN and CE geotypes of *N. commune* by biochemical and proteomic approach. Our work aimed to enhance the understanding of cyanobacterial adaptation mechanisms to environmental stress by investigation of physiological parameters (phenolics, carotenoids and phycobiliproteins) and protein expression.

Our main goal in this study was to conduct a biochemical and proteomic analysis, hypothesizing the physiological feature differences in two distant geotypes, particularly emphasizing protein expression in both AN and CE accessions of *N. commune*. This molecular study aimed to enhance our understanding of cyanobacterial physiology and their adaptation mechanisms to environmental stress.

## 2. Material and methods

### 2.1. Collection of plant material

Both *N. commune* samples were collected in the local springtime during fresh and sunny day. The vouchers are kept in the cryptogam collection of the herbarium of Pavol Jozef Šafárik University in Košice (KO), s/n. CE sample, leg./det. Martin Bačkor, 5. May 2017 Illmitz surrounding, Austria (47° 45′ 26″ S, 16° 49′ 01″ E), 134 m a.s.l., on bare soil in salty grassland; AN sample leg./det. Martin Bačkor, 12. January 2017 James Ross Island, near to Czech Polar Station of J.G. Mendel, ANa (63° 48′ 02″ S 57° 52′ 57″ E), 91 m a.s.l., on bare mineral rich substrate. Identification was done by Prof. Bačkor.^19–21^

### 2.2. Physiological and morphological characteristics

#### 2.2.1. Heterocyst count and length

The images and data of *N. commune* were adapted from the research of Ručová *et al*.^8^ A minimum of 200 heterocysts from both ecotypes of *N. commune* were included for examination. Original images for the above mentioned research were taken at Cell imaging and Ultrastructure Research, University of Vienna, Austria using OlympusBX41, camera Olympus U-CMAD3. Heterocyst length was determined using ImageJ software, with 50 heterocysts sampled from each *N. commune* type.

#### 2.2.2. Phenol content

Total phenol content in *N. commune* extracts was determined with Folin-Ciocalteu (FC) reagent.^22^ We have slightly modified FC reagent and used gallic acid as standard. Mixture of 100 µl of *N. commune* extract, 250 µl FC reagent, and 1 ml aqueous 20% Na_2_CO_3_ solution were prepared and incubated for 15 min at room temperature. The absorbance was measured by spectrophotometer (multi-detection micro plate reader; Synergy HT, Biotek) at 650 nm using MeOH as blank. The total phenol content was determined as the equivalent of gallic acid (in microgram) by the equation (*n* = 5):

Total phenols = [(A_650_ − 0.1059)/0.0525] µg g^−1^

[which was obtained by the calibration curve of Gallic acid (R^2^ = 0.9647)]

#### 2.2.3. Flavonoids

The content of flavonoids in the samples of *N. commune* was determined by colorimetry using rutin as standard.^23^ Solution of 2% methanol AlCl_3_ was prepared. Combination of 1 ml of MeOH extracts of *N. commune* and 1 ml of AlCl_3_ solution was mixed thoroughly and incubated at room temperature for 10 min. Absorbance was measured in spectrophotometer (multi-detection micro plate reader; Synergy HT, Biotek) at 415 nm with MeOH as blank. The total flavonoid content was determined as the equivalent of rutin (in µg) by using the following equation (*n* = 5):

Total flavonoids = [(A_415_ +1.028)/0.218] µg g^−1^

[Which was obtained from the standard graph of rutin (R^2^ = 0.9921)].

#### 2.2.4. Carotenoids

Carotenoid content was determined according to modified method described by Wellburn.^24^ Approximately, 4 mg of DW of *N. commune* samples were extracted in 1 ml of dimethyl sulfoxide (DMSO) at 65°C in dark for 1 h. After incubation, it was allowed cool down to room temperature and the absorbance of extracts were measured at 665, 649, 647, 663, 537 and 480 nm using spectrophotometer (multi-detection micro plate reader; Synergy HT, Biotek). The content of carotenoid was obtained at 480 nm and calculated by following equation,^24^ *n* = 5.

Total carotenoids = [(1000 × A_480_ − 2.14 × Content of Chl.a − 70.16 × Content of Chl.b)/220] mg ml^−1^

#### 2.2.5. Phycobilliproteins

For measurement of phycobiliproteins [Phycoerythrin (PE), phycocyanin (PC), allophycocyanin (APC)], 150 mg of *N. commune* samples were homogenized using 5 ml of DMSO and centrifuged at 2000 rpm for 5 min. The supernatant was obtained and measured at 562 nm for PE, 615 nm for PC and 652 nm for APC by spectrophotometer (multi-detection micro plate reader; Synergy HT, Biotek). The quantity of phycobiliproteins content was calculated by using the following equations,^25,26^ *n* = 5.

$$\mathrm{APC}=\;\left[\left(A_{652}-0.208\times A_{615}\right)/5.09\right]\mathrm{mg}\;ml^{-1}$$

$$\mathrm{PC}\;=\left[\left(A_{615}-0.474\times A_{652}\right)/5.34\right]\mathrm{mg}\;ml^{-1}$$

$$\mathrm{PE}\;=\left[\left(A_{562}-2.41\times \;\mathrm{PC}\;-\;0.849\times \mathrm{APC}\right)/9.62\right]\mathrm{mg}\;ml^{-1}$$

#### 2.2.6. Statistics

Statistical analyses were done with the software MINITAB 18. Significant differences were determined by one-way ANOVA and Tukey’s pairwise comparison of the mean. Values are given in average mean ± standard deviation (SD). Significant differences are denoted by letters “a” and “b”, with “n” representing the number of replications. Groups of samples sharing the same letter indicate no significant differences.

### 2.3. Proteomic analysis

For proteomic analysis, different geotypes (three biological replicates each) were freeze-dried in liquid N_2_ and ground for 2 min in a shaking mill using steel balls (2 mm diameter). The proteins were extracted, pre-fractionated (40 µg of total protein were loaded on to the gel (1D SDS-PAGE)), trypsin digested and desalted (using a C18 spec plate) according to a previously described method.^12,27,28^ Prior to mass spectrometric measurement, the tryptic peptide pellets were dissolved in 4% (v/v) acetonitrile, 0.1% (v/v) formic acid. One µg of each sample (three replicates for each geotype) was loaded on a C18 reverse-phase column (Thermo scientific, EASY-Spray 500 mm, 2 µm particle size). The separation was achieved with a 90 min gradient from 98% solution A (0.1% formic acid) and 2% solution B (90% acetonitrile and 0.1% formic acid) at 0 min to 40% solution B at 90 min with a flow rate of 300 nL min^−1^. Nano-electrospray ionization-MS/MS measurements were performed on an Orbitrap Elite (Thermo Fisher Scientific, Bremen, Germany) with the following settings: full scan range 350–1800 m/z, resolution 120 000, max. 20 MS2 scans (activation type CID), repeat count 1, repeat duration 30 s, exclusion list size 500, exclusion duration 30 s, charge state screening enabled with the rejection of unassigned and +1 charge states, and minimum signal threshold 500.

#### 2.3.1. Peptide and protein identification

Raw data were searched with the SEQUEST algorithm present in Proteome Discoverer version 1.3 (Thermo, Germany) described previously.^29,30^ The UniProt database for *N. commune* was used for identification of the proteins including biological function/processes (Supplementary table S1). Peptides were matched against these databases plus decoys, considering a significant hit when the peptide confidence was high, which is equivalent to a false discovery rate (FDR) of 1%, and the Xcorr threshold was established at 1 per charge (2 for + 2 ions 3 for + 3 ions, etc.). The variable modifications were set to acetylation of the N-terminus and methionine oxidation, with a mass tolerance of 10 ppm for the parent ion and 0.8 Da for the fragment ion. The number of missed and nonspecific cleavages permitted was two. There were no fixed modifications, as dynamic modifications were used. The identified proteins were quantitated based on total ion count and normalized using the normalized spectral abundance factor (NSAF) strategy.^31^

### 2.4. Bioinformatics and statistics for proteome data analysis

Raw files were processed in Proteome Discover (v1.3) (Thermo, Germany). Statistical and bioinformatic analysis were performed using ProVision (https://provision.shinyapps.io/provision/), a web-tool. For proteomics data, heatmap and principal component analysis (PCA) was computed and constructed using the R software. Venn diagram was generated using free online software (https://bioinformatics.psb.ugent.be/webtools/Venn/). For functional annotation of proteins UniPort Knowledgebase was used.

## 3. Results

The comparative analysis of cyanobacteria *N. commune* from two distinct geotypes, AN and CE revealed significant differences in their physiological and molecular functions. Geotype AN exhibited higher content carotenoid, phenol and flavonoids compared to CE geotype. In addition, proteomic analysis further identified distinct protein variations between the two geotypes, suggesting adaptation to their respective environmental conditions. A comprehensive analysis of the results is presented in the following subsections, offering detailed explanations and specific findings related to the study.

### 3.1. Microscopic analyses

**Figure 1.**
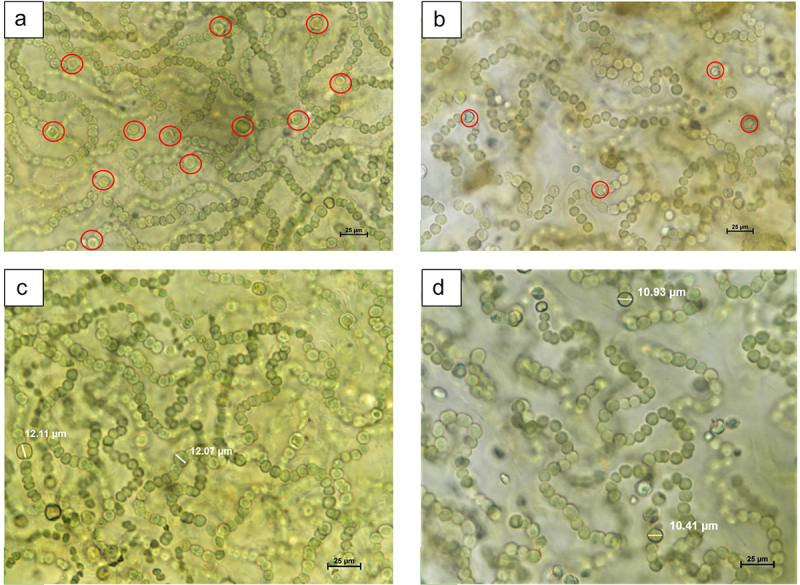
(A-D): N. commune heterocysts : AN geotype displaying numerous heterocysts (a) compared to the CE geotype with fewer heterocysts (b). Heterocyst length comparison between an (c) and CE (d) geotypes.

*N*. *commune* is a filamentous cyanobacteria that exists in macroscopic colonies. *N. commune* has different cell types: vegetative or photosynthetic cells for carbon fixation and heterocysts for nitrogen fixation. However, the number and length of this heterocyst varies due to the environmental conditions. Higher number of heterocyst (Avg. 7) was observed in AN *N. commune*, that is approximately 1.5 times more than CE (Avg. 4) *N. commune*. The mean length of heterocyst in *N. commune* colonies from AN was 11.793 ± 0.802 μm. In contrast, in CE *N. commune*, we observed smaller cells with a mean length of 10.6568 ± 0.6677 μm (Figure 1(a-d)). The study was adapted from Ručová *et al*.^8^

### 3.2. Total phenol and flavonoid content

**Figure 2.**
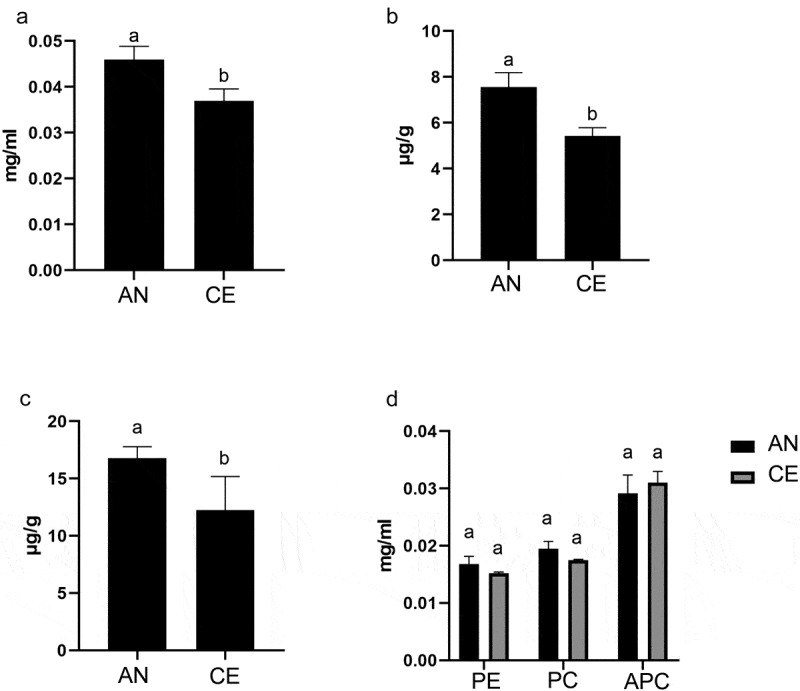
(A-D) Compositional parameter analysis between an and CE of N. commune. Content of (a) carotenoid, (b) flavonoid, (c) phenols and (d) phycobiliproteins.

Our study showed the difference in the total content of Phenols and Flavonoids of *N. commune* from different geotype (Figure 2(b,c)). In AN geotype, the total phenol and flavonoids content were 16.77 µg g^−1^ and 7.564 µg g^−1^, respectively, *n* = 5. In CE geotype, total phenol and flavonoids were 12.24 µg g^−1^ and 5.417 µg g^−1^, respectively, *n* = 5. Significant differences were observed between AN and CE *N. commune*.

### 3.3. Total carotenoid and phycobiliprotein content

We found total carotenoid content in AN geotype was 0.046 mg ml^−1^ or 0.011 µg mg^−1^ and CE geotype was 0.0369 mg ml^−1^ or 0.009 µg mg^−1^, n = 5 (Figure 2(a)), where *p* value = 0.001 with a significance difference. Phycobiliproteins consist of phycocyanin (PC), phycoerythrin (PE), and allophycocyanin (APC). We observed that PC in AN geotype was 0.0194 mg ml^−1^ and CE geotype was 0.0174 mg ml^−1^. PE value was 0.168 mg ml^−1^ and 0.152 mg ml^−1^, and APC value was 0.0291 mg ml^−1^ and 0.31 mg ml^−1^ in CE and AN geotype, respectively (Figure 2d). The *p* value for PE was 0.109, PC was 0.056, and APC was 0.439. The PC and PE content showed a slight increase, although the difference was not statistically significant.

### 3.4. Protein LC-MS analysis

**Figure 3.**
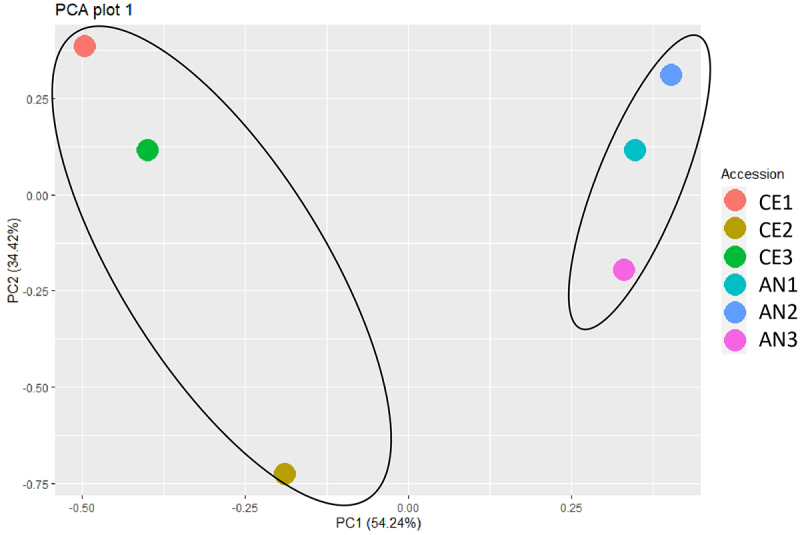
PCA plot derived from proteome data illustrates sample relationships. Points represent samples, reflecting proteomic profile similarities among N. commune from both geotype.

Investigation of the proteome responses of *N. commune* in different geotypes was performed using a LC-MS analysis. In total, 1147 proteins were quantified. The detailed information of identified proteins is available in supplementary Table S1. Six hundred and forty six significant proteins were identified which were up regulated in both the geotypes. The Principal component analysis (PCA) revealed the variation between the two geotypes at PC1 with a variation of 54.24% (Figure 3).

**Figure 4.**
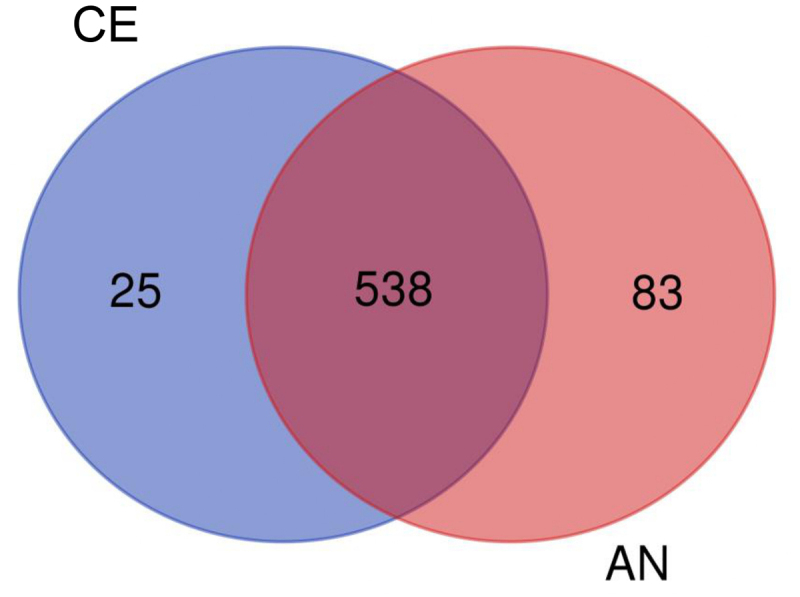
Differentially expressed proteins in two N. commune geotypes (common and exclusive) represented by Venn diagram.

The Venn analysis provided a clear distribution of proteins among the two different geotypes (Figure 4). From the significant 646 proteins, 538 proteins were common in both the CE and AN geotypes. Apart from the common proteins, 83 unique proteins were identified in the AN geotype. In contrast 25 unique proteins were identified in CE geotype, indicating molecular changes at the cellular level affecting their physiology, which is consistent with our results of physiological observation.

**Figure 5.**
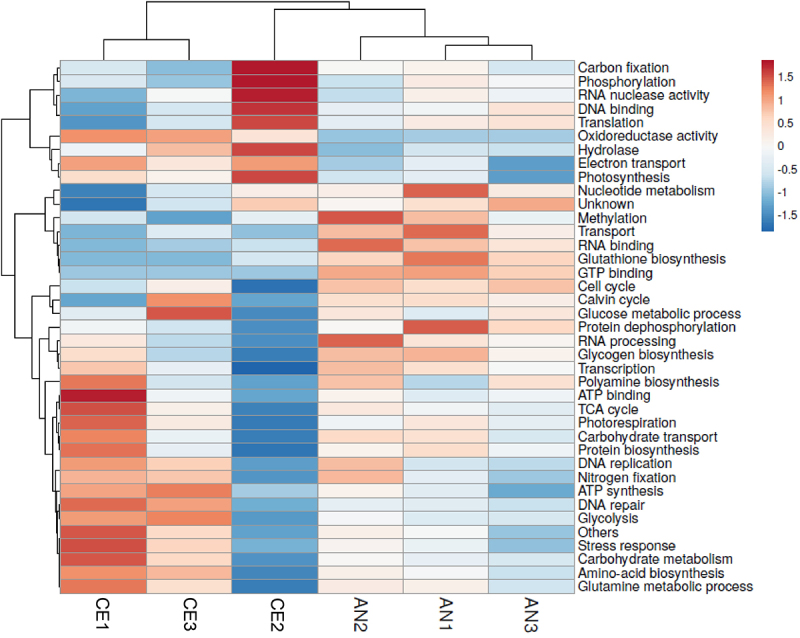
Heat map of N. commune proteomics showing various protein groups showing difference in protein up regulation within replicas between two geotypes. The hierarchical clustering was performed based on the Pearson’s correlation coefficients.

The identified 646 proteins were grouped into separate functional categories using UniPort Knowledgebase (https://www.uniprot.org/uniprotkb/) annotations and clustered into 58 groups (supplementary Table S1). A heat map, illustrated in (Figure 5), was generated to visually represent the regulatory patterns of proteins in two distinct geotypes. This analysis facilitated the comprehension of protein expression variations under distinct environmental conditions. Additionally, functional annotation of proteins provided insights into the explanation of biological processes affected by environmental changes, offering a comprehensive view of the molecular responses in each geotype. Analysis of all the significant proteins revealed that approximately 6.1% were functionally associated with photosynthesis and carbon fixation. Another substantial cluster of proteins played roles in carbohydrate metabolism, protein translation, amino acid biosynthesis, and proteolysis. Proteins participating in energy and primary metabolism, including the Calvin cycle, carbohydrate transport, biosynthesis processes such as porphyrin and polyamine biosynthesis, glucose metabolic processes, and protein biosynthesis, exhibited enhanced regulation in AN geotype. Furthermore, proteins associated with DNA binding, RNA binding, FAD binding, GTP binding, glutathione biosynthesis, and translation displayed higher regulation in the AN geotype compared to the CE geotype. However, proteins associated with photosynthesis, photorespiration, glycolysis, electron transport, nitrogen fixation were highly regulated in CE geotype. The nitrogen fixation protein results are persistent with the higher content of nitrogen in CE *N. commune*. Proteins related to water stress (A0A2Z6E9I3, A0A2Z6E9H8, A0A2Z6E9H7, T1SWJ9, F2Z6W9) were expressed, although their specific functions remain unknown.

Proteins such as Superoxide dismutase and Glutathione reductase (GRase) were explicitly expressed AN geotype indicating environmental challenges to grow and thrive. Similarly, small GTP-binding protein (A0A2R5FPH3), Glycolate oxidase subunit GlcD (A0A2R5FVB8), SpoIID/LytB domain-containing protein (A0A2R5FFL7) responsible for GTP binding, FAD binding and cellular spore formation respectively were only expressed in AN geotype.

In contrast to the above observation, there were not so many proteins identified in CE *N. commune*, which can be correlated to their physiological variations with the environment. However, our proteomics study confirms our hypothesis that *N. commune* growing in Antarctica can endure severe climatic conditions through robust protein regulation. A comparable response was not evident in the CE geotype.

## 4. Discussion

Cyanobacteria, including *N. commune*, belong to the most primitive life form on Earth. They are well adapted to several ecosystems including Antarctic habitats. A comparison of morphological and physiological characteristics confirms the aberration according to the geotype.^8^ However, the heterocyst production depends on the availability of N_2_ limitation. Since there is less availability of nitrogen resources at the collection area of *N. commune* colonies of Antarctica, higher numbers and bigger heterocysts were observed. Studies in cyanobacteria reveal in order to resist the damaging effect of UV radiation, they exhibit several strategies like mat formation, synthesis of phenolic compounds, proteins, carotenoid and scytonemin.^32,33^ Carotenoids, as auxiliary pigments in photosynthesis, protect plants by converting the excitation energy of triplet-state chlorophyll or de-exciting singlet oxygen, mitigating potential photo-oxidative damage.^34^ Likewise, scytonemin, a compound with photoprotective quality, protecting cyanobacteria from high UV radiation. Higher content of these compounds in the AN geotype was also observed in the latest study (unpublished data). This study further explains the environmental hazard on the *N. commune* in Antarctica rather than Europe. Similarly, our result demonstrated that an increase in the amount of carotenoids also provides evidence of environmental stress. Phycobiliproteins (PBPs), water-soluble pigment proteins identified in cyanobacteria like *Synechocystis* and *Mastigoclaudus laminosus*, constitute the primary components of the light-harvesting complex.^35^ The abundance of phycobiliproteins can be altered in response to environmental stress.^36^ The accumulation of higher levels of PC in AN *N. commune* than in the CE geotype confirms the survivability in harsher environmental conditions.

Proteomics analysis provided a clear image to understand the molecular regulation of cyanobacteria that can be well correlated to the physiological response. This study offers foundational evidence of the *N. commune* proteome, which can be a useful resource for future research. Functional grouping of proteins enlightens biological insights into cyanobacterial physiology and adaptation. In *N. commune* total 646 significant proteins were filtered which are differentially expressed. Considering the lack of existing protein functional annotation in *N. commune*, our focus is to explore a few well-documented proteins within cyanobacteria that hold significance for our study.

Photosynthesis and respiration are fundamental factors in energy metabolism process of photoautotrophs, which includes cyanobacteria. Study of *Nostoc* desiccation tolerance explained the dehydration and rehydration time period are directly proportional to each other and at proteome level, a substantial amount of proteins were involved in carbohydrate and energy metabolism.^37^ In the present study, we identified large number of proteins, which were involved in carbohydrate metabolism and photosynthesis. However, there regulation was not significant between both geotypes of *N. commune*.

Under stress conditions, cyanobacteria prevent the production of reactive oxygen species (ROS). Study on *N. commune* DRH1 under UV-B radiation-induced superoxide radicals generation and scavenged by SOD located within glycan.^38^ Superoxide dismutase (A0A193PSL3) known as one of the most important metalloenzymes during oxidative stress, and in our study identification of SOD in AN *N. commune* samples indicates adaptation and defense system against oxidative stress triggered by environment. Glutathione reductase (A0A2R5FMW0) an essential enzyme which catalyses the conversion of glutathione disulfide (GSSG) to reduced form Glutathione (GSH) in the presence of NADPH and belongs to pyridine-nucleotide disulfide oxidoreductase family of flavoenzymes, they protect against free radical and ROS.^39^ Glutathione synthase (GS) (A0A2R5FNB9), being an important enzyme in the ammonium assimilation pathway, is closely related to nitrogen fixation in cyanobacteria.^40^ Regulation of this enzyme in AN samples clearly imply the adaptation mechanism of the cyanobacteria. In the proteome study of *N. punctiforme*, significant amount of proteins were detected associated with porphyrin, phycobiliproteins, chlorophyll a and major photosynthetic light-harvesting pigments. In addition to the above, higher expression of superoxide dismutase, peroxidase, peroxiredoxin and DNA-binding ferritin-like proteins were detected in AN *N. commune*, which may play an important role in oxidative stress.^41^

Membrane transporter proteins are strong indicators of nutrient and ion exchange. They are very crucial in cyanobacteria adaptation to environmental stress. In abiotic stress conditions, sulfur metabolism activates various adaptive mechanisms.^42^ Bicarbonate (HCO_3_^−^) plays an important role in the maintenance of cellular pH. Additionally, several proteins with transport functions were identified with phosphorylation such as ammonium transporter and potassium-transporting ATPase.^43^ In our research, we identified the expression of several transport proteins and a higher number of transporter proteins in AN *N. commune* which might be due the habitat and growth conditions.

Mycosporine-like amino acids (MAA) synthesis was induced by water stress protein. In addition, water stress protein plays a vital role in synthesizing oligosaccharide-MAA due to UV stress.^18^ However, in the group of unknown proteins we observed several water stress proteins but further studies are required to unravel their specific function and direct involvement in the synthesis of MAA. Fatty acid and lipid compositions were altered as a response to UV stress and to counteract the damage *N. commune* expressed the related proteins. Our study also identified that homologues fatty acid synthesis proteins might be induced due to environmental challenges.

FAD binding proteins plays a vital role such as electron transport and photosynthesis, redox reaction, stress responses, signal transduction, etc. GTP binding proteins are important molecular switches involved in translation, ribosome biogenesis, cell division, maintenance of cellular homeostasis, RNA processing and stress responses. Interestingly, our proteome study revealed their explicit regulation of the proteome in AN *N. commune*, reflecting their survival ability in harsh environmental conditions, including physiological modifications compared to CE *N. commune*. In our knowledge, this is the first proteomic analysis of *N. commune* adaptation toward abiotic stress and further studies in climate adaptation of this species also needs to be investigated.

## 5. Conclusion

This study contributes toward comprehensive analysis of *N. commune* cyanobacteria’s adaptation and physiological characteristics in different environmental conditions. The results clearly demonstrate that *N. commune* from Antarctica is subjected to more environmental challenges. Our study brings the first insight into the proteomics of this cyanobacteria, also elucidating the protein expression under different condition. Furthermore, it suggests high adaptive amplitude of *N. commune*.

## Supplementary Material

Supplementary_table1.xlsx

## Data Availability

The data presented in this study can be provided by the authors upon reasonable request.

## References

[1] Schad M, Konhauser KO, Sánchez-Baracaldo P, Kappler A, Bryce C. How did the evolution of oxygenic photosynthesis influence the temporal and spatial development of the microbial iron cycle on ancient Earth? Free Radical Biol Med. 2019;140:154–9. doi:10.1016/j.freeradbiomed.2019.07.014. 31323314

[2] Sound JK, Bellamy-Carter J, Leney AC, Britt H, Beveridge R, Calabrese A. The increasing role of structural proteomics in cyanobacteria. Essays Biochem. 2023;67(2):269–282. doi:10.1042/EBC20220095. 36503929 PMC10070481

[3] Babele PK, Kumar J, Chaturvedi V. Proteomic de-regulation in cyanobacteria in response to abiotic stresses. Front Microbiol. 2019;10:1315. doi:10.3389/fmicb.2019.01315. 31263458 PMC6584798

[4] Holst J, Butterbach-Bahl K, Liu C, Zheng X, Kaiser AJ, Schnitzler J-P, Zechmeister-Boltenstern S, Brüggemann N. Dinitrogen fixation by biological soil crusts in an Inner Mongolian steppe. Biol Fertil Soils. 2009;45(7):679–690. doi:10.1007/s00374-009-0378-7.

[5] Novis PM, Whitehead D, Gregorich EG, Hunt JE, Sparrow AD, Hopkins DW, Elberling B, Greenfield LG. Annual carbon fixation in terrestrial populations of Nostoc commune (Cyanobacteria) from an Antarctic dry valley is driven by temperature regime. Global Change Biol. 2007;13(6):1224–1237. doi:10.1111/j.1365-2486.2007.01354.x.

[6] Sand-Jensen K, Jespersen TS. Tolerance of the widespread cyanobacterium Nostoc commune to extreme temperature variations (−269 to 105°C), pH and salt stress. Oecologia. 2012;169(2):331–339. doi:10.1007/s00442-011-2200-0. 22120705

[7] Scherer S, Ernst A, Chen T-W, Böger P. Rewetting of drought-resistant blue-green algae: time course of water uptake and reappearance of respiration, photosynthesis, and nitrogen fixation. Oecologia. 1984;62(3):418–423. doi:10.1007/BF00384277. 28310898

[8] Ručová D, Goga M, Matik M, Bačkor M. Adaptations of cyanobacterium Nostoc commune to environmental stress: comparison of morphological and physiological markers between European and Antarctic populations after rehydration. Czech Polar Rep. 2018;8(1):84–93. doi:10.5817/CPR2018-1-6.

[9] Diane Boudreau MM, Sprout E, Turgeon A. Europe: resources. Washington DC: National Geographic Society; 2023.

[10] Weckwerth W, Ghatak A, Bellaire A, Chaturvedi P, Varshney RK. PANOMICS meets germplasm. Plant Biotechnol J. 2020;18(7):1507–1525. doi:10.1111/pbi.13372. 32163658 PMC7292548

[11] Battchikova N, Muth-Pawlak D, Aro E-M. Proteomics of cyanobacteria: current horizons. Curr Opin Biotechnol. 2018;54:65–71. doi:10.1016/j.copbio.2018.02.012. 29499477

[12] Valledor L, Weckwerth W. An improved detergent-compatible gel-fractionation LC-LTQ-Orbitrap-MS workflow for plant and microbial proteomics. Methods Mol biol (Clifton, NJ). 2014;1072:347–358. doi:10.1007/978-1-62703-631-3_25. 24136534

[13] Mann M, Hendrickson RC, Pandey A. Analysis of proteins and proteomes by mass spectrometry. Annu Rev Biochem. 2001;70(1):437–473. doi:10.1146/annurev.biochem.70.1.437. 11395414

[14] Andrews GL, Simons BL, Young JB, Hawkridge AM, Muddiman DC. Performance characteristics of a new hybrid quadrupole time-of-flight tandem mass spectrometer (TripleTOF 5600). Anal Chem. 2011;83(13):5442–5446. doi:10.1021/ac200812d. 21619048 PMC3138073

[15] Eliuk S, Makarov A. Evolution of orbitrap mass spectrometry instrumentation. Annu Rev Anal Chem (Palo Alto Calif). 2015;8(1):61–80. doi:10.1146/annurev-anchem-071114-040325. 26161972

[16] Hu Q, Noll RJ, Li H, Makarov A, Hardman M, Graham Cooks R. The Orbitrap: a new mass spectrometer. J Mass Spectrom. 2005;40(4):430–443. doi:10.1002/jms.856. 15838939

[17] Koksharova OA, Butenko IO, Pobeguts OV, Safronova NA, Govorun VM. The first proteomic study of Nostoc sp. PCC 7120 exposed to cyanotoxin BMAA under nitrogen starvation. Toxins. 2020;12(5):310. doi:10.3390/toxins12050310. 32397431 PMC7290344

[18] Ehling‐Schulz M, Schulz S, Wait R, Görg A, Scherer S. The UV‐B stimulon of the terrestrial cyanobacterium Nostoc commune comprises early shock proteins and late acclimation proteins. Mol Microbiol. 2002;46(3):827–843. doi:10.1046/j.1365-2958.2002.03209.x. 12410839

[19] Hindák F. Colour Atlas of cyanophytes. VEDA; 2008. p. 253. ISBN 9788022410441.

[20] Komárek J, Elster J. Ecological background of cyanobacterial assemblages of the northern part of James Ross Island, Antarctica. Pol Polar Res. 2008;29(1):17–32.

[21] Skácelová K, Hrbáček F, Chattová B, Láska K, Barták M. Biodiversity of freshwater autotrophs in selected wet places in northern coastal ecosystems of James Ross Island. Czech Polar Rep. 2015;5(1):12–26. doi:10.5817/CPR2015-1-2.

[22] Ghatak AA, Chaturvedi PA, Desai NS. Indian grape wines: a potential source of phenols, polyphenols, and antioxidants. Int J Food Prop. 2014;17(4):818–828. doi:10.1080/10942912.2012.675608.

[23] Meda A, Lamien CE, Romito M, Millogo J, Nacoulma OG. Determination of the total phenolic, flavonoid and proline contents in Burkina Fasan honey, as well as their radical scavenging activity. Food Chem. 2005;91(3):571–577. doi:10.1016/j.foodchem.2004.10.006.

[24] Wellburn AR. The spectral determination of chlorophylls a and b, as well as total carotenoids, using various solvents with spectrophotometers of different resolution. J Plant Physiol. 1994;144(3):307–313. doi:10.1016/S0176-1617(11)81192-2.

[25] Bennett A, Bogorad L. Complementary chromatic adaptation in a filamentous blue-green alga. J Cell Biol. 1973;58(2):419–435. doi:10.1083/jcb.58.2.419. 4199659 PMC2109051

[26] Bryant DA, Guglielmi G, de Marsac NT, Castets A-M, Cohen-Bazire G. The structure of cyanobacterial phycobilisomes: a model. Arch Microbiol. 1979;123(2):113–127. doi:10.1007/BF00446810.

[27] Chaturvedi P, Ischebeck T, Egelhofer V, Lichtscheidl I, Weckwerth W. Cell-specific analysis of the tomato pollen proteome from pollen mother cell to mature pollen provides evidence for developmental priming. J Proteome Res. 2013;12(11):4892–4903. doi:10.1021/pr400197p. 23731163

[28] Ghatak A, Chaturvedi P, Nagler M, Roustan V, Lyon D, Bachmann G, Postl W, Schröfl A, Desai N, Varshney RK. et al. Comprehensive tissue-specific proteome analysis of drought stress responses in Pennisetum glaucum (L.) R. Br. (Pearl millet). J Proteomics. 2016;143:122–135. doi:10.1016/j.jprot.2016.02.032. 26944736

[29] Chaturvedi P, Doerfler H, Jegadeesan S, Ghatak A, Pressman E, Castillejo MA, Wienkoop S, Egelhofer V, Firon N, Weckwerth W. Heat-treatment-responsive proteins in different developmental stages of tomato pollen detected by targeted Mass Accuracy Precursor Alignment (tMAPA). J Proteome Res. 2015;14(11):4463–4471. doi:10.1021/pr501240n. 26419256

[30] Ghatak A, Chaturvedi P, Bachmann G, Valledor L, Ramšak Ž, Bazargani MM, Bajaj P, Jegadeesan S, Li W, Sun X. et al. Physiological and proteomic signatures reveal mechanisms of superior drought resilience in Pearl Millet Compared to Wheat. Front Plant Sci. 2020;11:600278. doi:10.3389/fpls.2020.600278. 33519854 PMC7838129

[31] Paoletti AC, Parmely TJ, Tomomori-Sato C, Sato S, Zhu D, Conaway RC, Conaway JW, Florens L, Washburn MP. Quantitative proteomic analysis of distinct mammalian Mediator complexes using normalized spectral abundance factors. Proc Natl Acad Sci USA. 2006;103(50):18928–18933. doi:10.1073/pnas.0606379103. 17138671 PMC1672612

[32] Rastogi RP, Kumari S, Richa R, Han T, Sinha RP. Molecular characterization of hot spring cyanobacteria and evaluation of their photoprotective compounds. Can J Microbiol. 2012;58(6):719–727. doi:10.1139/w2012-044. 22582897

[33] Singh SP, Häder DP, Sinha RP. Cyanobacteria and ultraviolet radiation (UVR) stress: mitigation strategies. Ageing Res Rev. 2010;9(2):79–90. doi:10.1016/j.arr.2009.05.004. 19524071

[34] Di Mascio P, Kaiser S, Sies H. Lycopene as the most efficient biological carotenoid singlet oxygen quencher. Arch Biochem Biophys. 1989;274(2):532–538. doi:10.1016/0003-9861(89)90467-0. 2802626

[35] Sinha R, Kumar A, Tyagi M, Häder D. Ultraviolet-B-induced destruction of phycobiliproteins in cyanobacteria. Physiol Mol Biol Plants. 2005;11:313–319.

[36] Hemlata H, Fatma T. Screening of cyanobacteria for phycobiliproteins and effect of different environmental stress on its yield. Bull Environ Contam Toxicol. 2009;83(4):509–515. doi:10.1007/s00128-009-9837-y. 19629363

[37] Liang W, Zhou Y, Wang L, You X, Zhang Y, Cheng CL, Chen W. Ultrastructural, physiological and proteomic analysis of Nostoc flagelliforme in response to dehydration and rehydration. J Proteomics. 2012;75(18):5604–5627. doi:10.1016/j.jprot.2012.07.041. 22884584

[38] Shirkey B, Kovarcik DP, Wright DJ, Wilmoth G, Prickett TF, Helm RF, Gregory EM, Potts M. Active Fe-containing superoxide dismutase and abundant sodF mRNA in Nostoc commune (Cyanobacteria) after years of desiccation. J Bacteriol. 2000;182(1):189–197. doi:10.1128/jb.182.1.189-197.2000. 10613879 PMC94256

[39] Arias DG, Marquez VE, Beccaria AJ, Guerrero SA, Iglesias AA. Purification and characterization of a glutathione reductase from phaeodactylum tricornutum. Protist. 2010;161(1):91–101. doi:10.1016/j.protis.2009.06.001. 19664954

[40] Gao K. Chinese studies on the edible blue-green alga, Nostoc flagelliforme: a review. J Appl Phycol. 1998;10(1):37–49. doi:10.1023/A:1008014424247.

[41] Anderson DC, Campbell EL, Meeks JC. A soluble 3D LC/MS/MS proteome of the filamentous cyanobacterium Nostoc punctiforme. J Proteome Res. 2006;5(11):3096–3104. doi:10.1021/pr060272m. 17081061

[42] Cheng W, Zhang L, Jiao C, Su M, Yang T, Zhou L, Peng R, Wang R, Wang C. Hydrogen sulfide alleviates hypoxia-induced root tip death in Pisum sativum. Plant Physiol Bioch. 2013;70:278–286. doi:10.1016/j.plaphy.2013.05.042. 23800663

[43] Wang B, Yang J, Xu C, Yi L, Wan C. Dynamic expression of intra‐and extra‐cellular proteome and the influence of epiphytic bacteria for Nostoc flagelliforme in response to rehydration. Environ Microbiol. 2020;22(4):1251–1264. doi:10.1111/1462-2920.14931. 31997460

